# Impact of Selected Behavioral and Environmental Factors on the Antibiotic Therapy in Polish Children With Upper Respiratory Tract Infections

**DOI:** 10.3389/fped.2021.784265

**Published:** 2021-12-03

**Authors:** Katarzyna Ślęzak, Łukasz Dembiński, Artur Konefał, Mikołaj Dąbrowski, Artur Mazur, Małgorzata Peregud-Pogorzelska, Paweł Wawrykow, Dorota Konefał, Jarosław Peregud-Pogorzelski

**Affiliations:** ^1^Department of Pediatrics, Pediatric Oncology and Immunology, Pomeranian Medical University, Szczecin, Poland; ^2^Department of Pediatric Gastroenterology and Nutrition, Medical University of Warsaw, Warsaw, Poland; ^3^Instytute of Clinical Improvement, Warsaw, Poland; ^4^Adult Spine Orthopaedics Department, Poznan University of Medical Sciences, Poznań, Poland; ^5^Department of Pediatrics, Pediatric Endocrinology and Diabetes, Medical College of Rzeszow University, Rzeszów, Poland; ^6^Department of Cardiology, Pomeranian Medical University, Szczecin, Poland

**Keywords:** antibiotic therapy, education, infectious diseases, pediatric primary care, vaccination

## Abstract

Antibiotic therapy must be carried out consistently and according to the guidelines. Viruses are the dominant cause of upper respiratory tract infections (URTIs) in children, as has been shown in many previous studies. Unnecessary antibiotic therapy should be avoided so that it does not affect patients' health and lead to the development of resistant bacterial strains. Here we report a national survey conducted in a group of 4,389 children to assess the impact of selected behavioral and environmental factors on antibiotic therapy in patients with URTIs. We found that selected environmental factors influenced the type of treatment. The place of residence, having siblings, an absence of vaccinations, the presence of allergies, and attendance at educational institutions were conducive to antibiotic therapy. These factors also influenced the frequency of hospitalization of children and their absence from nurseries, kindergartens, and schools, as well as the absence of their guardians from work.

## Introduction

Upper respiratory tract infections (URTIs) in children—define as infections of upper airways with no signs of pneumonia—are an important social and epidemiological problem. They are the most common reason for primary practice attendance in this group (1–3). The vast majority of URTIs are viral, including about 70–85% of pharyngitis cases and more than 90% of bronchitis cases (1–3). Bacterial infections are much less common and usually develop as a secondary infection (or superinfection) after a viral illness. It is essential that the proposed treatment for URTIs does not adversely affect the health of patients and prevents the development of bacterial strains that are resistant to antibiotics (4–6).

The duration of antibiotic therapy is usually <20 days (1). However, patients often fail to take their medication as prescribed, which can compromise therapeutic efficacy. Missing a dose of the drug is the most common issue. Additional doses are sometimes taken in the hope of accelerating recovery. Patients also may prematurely terminate their therapy in response to a resolution of symptoms or the occurrence of adverse effects, such as abdominal pain or diarrhea. Additionally, some patients delay starting their treatment in the hope that the infection will resolve spontaneously (7, 8). A European study showed that the patients expected clinical improvements to appear within 3 days of the start of antibiotic. This is likely to be one of the reasons for non-compliance with therapeutic regimens (8).

Antibiotic therapy must be adhered to consistently and following expert guidelines (1, 9). This is the only approach that will reduce the number of resistant bacterial strains and limit patient exposure to adverse effects and complications of the therapy. It also ensures the effective elimination of pathogenic microbes and controls the diseases that they cause (4, 10, 11). Due to the widespread abuse and misuse of antibiotics, many global and local guidelines for their proper use have been developed (1, 5, 6, 12–15). The rational use of antibiotics should lead to the rapid and complete elimination of pathogenic bacteria, with minimal adverse effects. Therefore, these agents must be used carefully, at adequate doses, and without treatment discontinuation before the end of the entire course of therapy (5, 13, 15). Preventive antimicrobial therapy in unjustified cases should be avoided, and the latest generation drugs should be used only for the most severe infections. Failure to follow these rules contributes to the increase in the number of drug-resistant strains. This, in turn, reduces the chances for recovery and necessitates the use of therapies that are associated with multiple adverse reactions (10, 16).

To explore this phenomenon, we conducted a non-interventional questionnaire study among the parents/guardians of 4,389 children treated for URTIs to investigate their preferred therapeutic regimens depending on the symptoms presented and the course of previous infections.

Furthermore, we examined the clinical and behavioral factors, and particularly the social and environmental issues, which had an impact on the choice of treatment strategy in children with URTIs in a primary practice setting, as well as parental compliance with medical recommendations.

## Aims

1. To Identify Clinical and Behavioral Factors Likely to Affect Antibiotic Therapy in Children With URTIs.
2. To Assess the Impact of Knowledge About Antimicrobial Treatment Among the Parents/Guardians of Children With URTIs on the Therapeutic Strategy Used.
3. To Determine Which of the Analyzed Factors and Behaviors of the Parents/Guardians of Children With URTIs Could Promote the Inclusion of Antibiotics by a General Practitioner (GP).

## Materials and Methods

This study had a questionnaire-based, observational, non-interventional design. No additional non-standard medical procedures were performed. The study was conducted in an outpatient setting (i.e., in primary health-care clinics) by pediatricians. Before inclusion in the study, the physician provided the patient with written information about the assumptions and goals of the program, and obtained oral consent from a parent/guardian for participation in the study. Each study participant attended a consultation during which information about the patient was collected (i.e., demographic and social data, and details of childhood infections).

The inclusion criteria were age <10 years and attendance of an appointment with a GP due to a URTI. The following exclusion criteria were used: chronic cough of unknown cause for >4 weeks; respiratory defects (e.g., tracheoesophageal fistula); gastroesophageal reflux disease; cystic fibrosis; chronic cardiovascular disease; asthma; immune deficiencies; immunosuppressant therapy; and fever >39°C.

As part of the study, each patient attended a GP visit during which their parent/guardian was asked to complete a questionnaire. This gathered patient's personal data and information about their current weight and birth weight, place of residence, exposure to tobacco smoke, attendance at a nursery, kindergarten or school, history of respiratory diseases, factors increasing the risk of infection, the number of prior infections, hospital stays, and missed days at nursery/kindergarten or school, and the number of symptoms suggesting primary immunodeficiency. Furthermore, it requested details of the symptoms of the infection that was the reason for the visit to the interviewing doctor and of the treatment used.

A test to check parental knowledge of antibiotic therapy (comprising the following five questions) was an important element of the questionnaire:

1. Is It Acceptable to use an Antibiotic That Has Been Left Over From Previous Treatment Without Consulting a Doctor?
2. Can an Antibiotic be Taken 1 h Before or 2 h After a Meal?
3. Is It Acceptable to Reduce or Increase the Dose of an Antibiotic Without Consulting a Doctor?
4. Should Probiotics be Taken During and After Antibiotic Therapy?
5. Is It Acceptable to Stop Antibiotic Therapy Earlier Than Recommended by the Doctor Once the Child Is Feeling Better?

Statistical analysis was performed using the Statistica 13.3 PL version (Statsoft, Tulsa, USA). The Kolmogorov–Smirnov test was applied to verify a normal distribution. The mean values of certain parameters were compared using the Student's *t*-test or the Mann–Whitney *U*-test for quantitative variables and the Chi-square (χ2) test for qualitative variables. The Kruskal–Wallis test was utilized for the analysis of variance. The correlation assessment was performed using the Spearman's rank correlation coefficient. Statistical significance was considered as *p* < 0.05. The data are presented as the mean value ± standard deviation (SD) or as a percentage of the patients in the analyzed groups. The values of the odds ratio (OR) and the 95% confidence interval (CI) were reported.

## Results

In total, 4,802 patients from 87 outpatient clinics in different country regions were enrolled in the study. Of these, 4,389 patients were included in the statistical analysis. Overall, 413 patients (8.6% of all those enrolled) were excluded due to incomplete data, failure to meet the inclusion criteria, or lack of parental/guardian consent.

The mean age of the children participating in the study was 4.9 years (median = 4.6 years), which fell between the lower quartile of 2.9 and the upper quartile of 6.6. The study group comprised 2,108 (48.0%) girls and 2,281 (52.0%) boys. There was no statistically significant difference between the genders in terms of age (*p* = 0.5). The girls had a significantly lower birth weight (*p* < 0.001).

As reported by the parents/guardians, URTIs affected 3,043 (69.3%) of the children. Chronic, including obstructive, bronchitis had developed in 555 (12.6%) cases and pneumonia had occurred in 539 (12.3%) of the patients during the previous year.

The most common symptoms of infections in these patients included the following: rhinitis in 3,262 (74.3%); cough in 2,938 (66.9%); decreased appetite in 2,869 (65.4%); diminished well-being in 2,042 (46.5%); reduced physical activity in 1,977 (45.0%); pharyngitis in 1,894 (43.2%); hoarseness in 1,471 (33.5%); and difficulty swallowing in 1,171 (26.7%). The mean duration of symptoms in the children attending medical appointments was 3.7 days, with a median of 3 days, which fell within the lower quartile of 2 and the upper quartile of 5. The mean number of symptoms was five and this value was significantly higher in the groups of children who had been exposed to tobacco smoke, attended an educational institution, had siblings, had been treated with antibiotics, and presented with fever (*p* < 0.01) (Table 1).

**Table 1:** The presence of at least five symptoms of URTIs and selected environmental and clinical factors in the study group (*n* = 4,389).

**Risk factors**	**Number of patients with <5 symptoms**	**Number of patients with ≥5 symptoms**	****χ^2^** test**
Cigarette-smoke exposure	347	617	***P*** **<** **0.01**
No exposure to cigarette smoke	1,595	1,830	
Siblings	822	1,152	
No siblings	1,120	1,295	***P*** **=** **0.02**
Daycare attendance	1,232	1,764	
No daycare attendance	710	683	***P*** **<** **0.01**
Normothermy	857	507	
Increased body temperature	1,085	1,940	***P*** **<** **0.01**
Antibiotic therapy	531	1,373	
No antibiotic	1,411	1,074	***P*** **<** **0.01**

**Figure 1:**
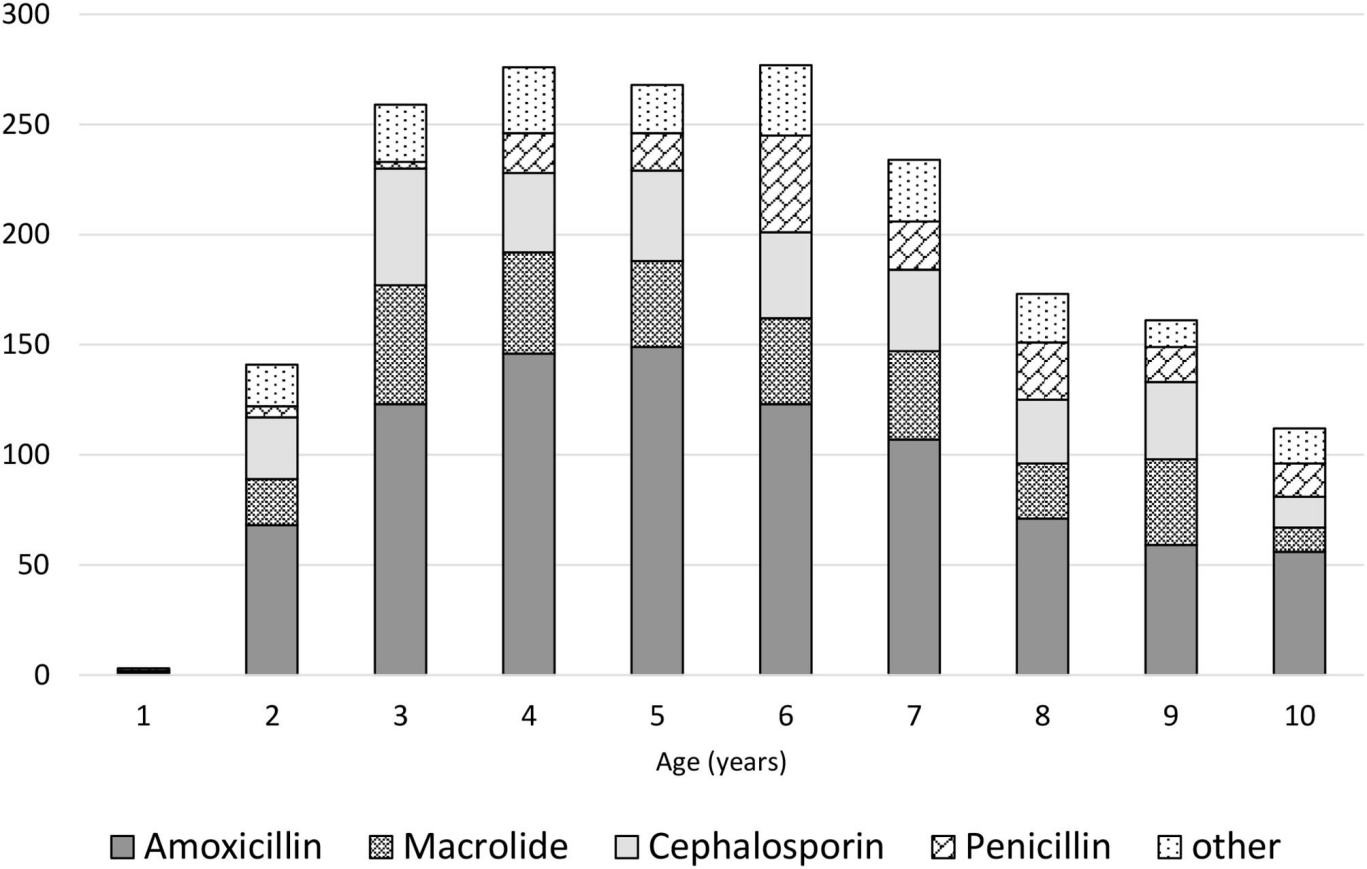
The types of antibiotics used depend on the age of the children.

Parents had administered treatments before the medical appointment in 84.1% of the children, including antipyretics/analgesics in 52.8% and nebulization in 30.8% of cases. During the medical appointment scheduled due to infection, anti-inflammatory drugs were recommended in 2,669 (60.9%) of the children, including the following: ibuprofen in 1,358 (50.8%) of the cases; paracetamol in 527 (19.7%); paracetamol with ibuprofen in 784 (29.4%); expectorants in 1,121 (25.5%); and inhaled steroids in 968 (22.1%). Antibiotics were used in 1,682 (38.3%) of all participants, including amoxicillin in 965 (57.4%) of the cases, macrolide in 345 (20.5%), and cephalosporin in 372 (22.1%). The relationship between the type of antibiotic and the age of children is presented in Figure 1. Probiotics were recommended for 2,486 (56.6%) of the participants. In 40.7% of cases, data on the probiotic strain were obtained, *Lactobacillus rhamnosus* (963, 37.7%) was dominant, and only in 0.9% *Saccharomyces boulardii* was used. Natural “immune-boosting” preparations were prescribed in 1,449 (33.0%) of the patients, with the most common being lactoferrin (488 cases or 33.7%) and herbal products e.g., elderberry and chokeberry extracts (269 cases or 18.6%).

More than 4,155 (85.9%) of the children had experienced an infection in the previous 6 months. The mean and median number of infections was 3. During the prior 6 months, antibiotic therapy had been used in 2,963 of the children, including once in 2/3 and twice in 1/5 of children. The number of hospitalizations was 713, which accounted for 16.2% of children in the study group. The mean age of the hospitalized children was statistically significantly lower than that of those treated on an outpatient basis, 5.0 and 4.2 years, respectively (*p* < 0.01). Despite the infections, 1,866 children (42.5%) had continued to attend their educational institutions throughout. Among those children who had stayed at home due to their infections, the mean duration of their absence was 20 days with a median of 15.

Antibiotic therapy was significantly more common in children living in rural areas (45.2 vs. 33.8%, χ^2^ test *p* < 0.01) and significantly less common in those residing in medium-sized municipalities (35.9 vs. 23.1%, χ^2^ test *p* < 0.01). Antibiotic therapy was significantly less often prescribed for children with siblings (46.4 vs. 43.1%, χ^2^ test *p* = 0.03). However, antibiotics were significantly more often prescribed for children with atopic diseases such as eczema or asthma (31 vs. 22.6%, χ^2^ test *p* < 0.01), those attending educational institutions (such as nurseries, kindergartens, and schools) compared to those being home schooled (χ^2^ test *p* < 0.001), and those who were exposed to tobacco smoke (31.5 vs. 23.8%, χ^2^ test *p* < 0.01) (Table 2). A statistically significant difference (*p* < 0.01) was found in the use of antibiotics depending on the type of feeding in infancy. The risk factor of antibiotic therapy was formula feeding compared to breastfeeding (48.4 vs. 34.4%).

**Table 2:** Antibiotic therapy and selected environmental factors in the study group (*n* = 4,389).

**Risk factor**	**Total number of patients**	**Antibiotic**	**No antibiotic**	**χ^2^ test**
Normal birth weight	4,236	1,838	2,398	
Low birth weight	153	66	87	*P* = 0.95
Male gender	2,281	1,020	1,261	
Female gender	2,108	884	1,224	*P* = 0.63
Siblings	1,974	821	1,153	
No siblings	2,415	1,083	1,332	***P*** **=** **0.03**
Vaccinated	4,271	1,851	2,420	
Non-vaccinated	118	53	65	*P* = 0.73
Allergy	1,151	590	561	
No allergy	3,238	1,314	1,924	***P*** **<** **0.01**
Breast-feeding	1,571	540	1,031	
Feeding with formula	2,818	1,364	1,454	***P*** **<** **0.01**
Towns with a population <10,000	560	265	295	***P*** **<** **0.01**
Towns with a population 10–100,000	1,333	440	895	
Countryside	1,700	860	840	
Cities with a population >100,000	796	339	457	
Countryside	1,700	860	840	***P*** **<** **0.01**

*Statistically significant p-values are shown in bold*.

No statistically significant differences were found in the frequency of antibiotic use depending on birth weight, preventive vaccinations, or gender. The number of antibiotic therapies used (*p* = 0.02) and the number of days missed from educational institutions (*p* < 0.01) significantly increased in children with elevated body temperature. The number of missed school/kindergarten days was significantly higher for children treated with natural “immune-boosting” preparations (*p* < 0.01). We also found that hospitalization rates were negatively correlated with age (Pearson's test *r* = 0.09, *p* = 0.02).

Having siblings, atopy, exposure to tobacco smoke, lack of vaccinations, attending nurseries or kindergartens, and living in rural areas or municipalities with a population of <10,000 inhabitants predisposed children to more frequent hospitalization with URTIs (Table 3).

**Table 3:** Hospitalization rates and selected environmental and clinical factors in the study group (*n* = 4,389).

**Risk factors**	**Total number of patients**	**Hospitalizations**	**Mean ± SD**	**Median (Q1–Q2)**	***p*-value**
Male gender	2,281	396	0.22 ± 0.95	0 (0–0)	
Female gender	2,108	317	0.19 ± 0.51	0 (0–0)	***P*** **=** **0.03**
Siblings	1,974	367	0.23 ± 0.56	0 (0–0)	
No siblings	2,415	346	0.18 ± 0.54	0 (0–0)	***P*** **<** **0.01**
Allergy	1,151	381	0.43 ± 0.70	0 (0–1)	
No allergy	3,238	332	0.13 ± 0.46	0 (0–0)	***P*** **<** **0.01**
Tobacco-smoke exposure	1,192	265	0.34 ± 0.68	0 (0–1)	
No tobacco-smoke exposure	3,197	448	0.15 ± 0.49	0 (0–0)	***P*** **<** **0.01**
Vaccinated	4,271	675	0.20 ± 0.54	0 (0–0)	
Non-vaccinated	118	38	0.47 ± 0.85	0 (0–1)	***P*** **<** **0.01**
Nursery attendance	561	185	0.44 ± 0.74	0 (0–1)	
Kindergarten attendance	1,842	262	0.18 ± 0.52	0 (0–0)	***P*** **<** **0.01**
School attendance	593	60	0.11 ± 0.35	0 (0–0)	
Home schooling	593	40	0.16 ± 0.43	0 (0–0)	***P*** **=** **0.04**
Kindergarten attendance	1,842	262	0.18 ± 0.52	0 (0–0)	
School attendance	593	60	0.11 ± 0.35	0 (0–0)	***P*** **<** **0.01**
Cities with a population <10,000	560	119	0.25 ± 0.56	0 (0–0)	
Cities with a population 10–100,000	1,333	124	0.11 ± 0.40	0 (0–0)	***P*** **<** **0.01**
Cities with a population 10–100,000	1,333	124	0.11 ± 0.40	0 (0–0)	
Countryside	1,700	382	0.29 ± 0.65	0 (0–0)	***P*** **<** **0.01**
Cities with a population >100,000	796	88	0.14 ± 0.51	0 (0–0)	
Countryside	1,700	382	0.29 ± 0.65	0 (0–0)	***P*** **<** **0.01**

The survey conducted among the parents/guardians of children with URTIs clearly showed that urban residents were significantly more likely (*p* < 0.01) than rural dwellers to provide accurate answers to questions about antibiotic therapy (Table 4).

**Table 4:** Responses to the knowledge survey.

**Question**	**Groups**	**Response**			
		**Yes**	**No**	**Undecided**	**Chi^**2**^ test**
Is it acceptable to use an antibiotic left over from a previous treatment without consulting a doctor?	Countryside	62	1,390	248	
	City	65	2,393	231	**<0.01**
Can an antibiotic be taken 1 h before or 2 h after a meal?	Countryside	942	258	500	
	City	1,509	381	799	<0.1
Is it acceptable to reduce or increase the dose of an antibiotic without consulting a doctor?	Countryside	40	1,475	185	
	City	47	2,545	97	**<0.01**
Can probiotics be taken during and after antibiotic therapy?	Countryside	1,227	174	198	
	City	2,309	182	198	**<0.01**
Is it acceptable to stop taking an antibiotic earlier than recommended by the doctor once a child is feeling better?	Countryside	85	1,387	228	
	City	136	2,383	170	**<0.01**

*Statistically significant p-values are shown in bold*.

## Discussion

To our knowledge, this is the first Polish non-interventional questionnaire study conducted in the developmental age population (represented by 4,389 patients) to assess the impact of selected environmental factors, the course of previous infections, manifestations, and parental knowledge of the use of antibiotic therapy in children with URTIs, as prescribed by GPs.

Among the assessed environmental, clinical, and social factors, the place of residence, having siblings, the presence of allergies, and children's attendance at educational institutions were found to be strong predictors of antibiotic therapy. They also influenced hospitalization rates and the absence of children from nurseries, kindergartens, and schools. We found that having siblings was a significant (*p* < 0.01) risk factor for respiratory infections (17–20). Previous research indicated that having siblings did not influence the severity of URTIs (21). This finding was confirmed by our observations. Antibiotic therapy was significantly less common among this group of patients (*p* < 0.01) (Table 1). This was probably due to increased parental experience of dealing with mild respiratory infections and additional knowledge acquired through previous pediatric medical appointments. Therefore, it seems important to improve parental education during medical visits for respiratory tract infections in children, which should in turn reduce the frequency of antibiotic use (22, 23).

Although the percentage of smokers has significantly decreased over past years, exposure to tobacco smoke remains a significant health problem (24). Many studies in large groups have confirmed that the risk (and severity) of respiratory tract infections, asthma, and other respiratory symptoms (such as cough, bronchial hyperreactivity, and recurrent infections) is increased in passive smokers (21, 25–28). In our study, the number of episodes of URTIs and antibiotic therapy was significantly higher in the group of children exposed to tobacco smoke (*p* < 0.01) (Table 1). The proportion of patients receiving antibiotics increased from 23.8% in children who were not exposed to tobacco smoke to 31.5% in those with chronic exposure. This clearly indicates the urgent need for intensive and extensive education for parents on the impact of tobacco smoke on the health of children.

Formula feeding in infancy proved to be a predictor of an increased frequency of antibiotic therapy. This is in line with the current observations on the positive impact of breastfeeding on the development of the immune system, and thus on the risk of infection and the frequency of antibiotic therapy (29–31). In the case of rural children, who had significantly lower rates of URTIs (*p* < 0.001) and higher rates of antibiotic therapy relative to the number of infections (*p* < 0.01), the limited availability of medical consultations played an important role: they tend to consult a doctor at a later stage of disease, when the need for antibiotics is more justified and more likely. Additionally, a lack of quick access to diagnostic tests, in the event of clinical doubts, affects the decision to initiate antibiotic treatment by the consulting physician, especially when the next follow-up visit is likely to be in the relatively distant future (32–34).

Undoubtedly, parental knowledge was an important predictor of the decisions made on the use of antibiotics in children with URTIs. We showed that urban dwellers were significantly more likely (*p* < 0.01) to correctly answer the questions about the use an antibiotics that had been left over from a previous treatment; the ingestion of an antibiotic 1 h before or 2 h after a meal; reducing or increasing the dose of an antibiotic without consulting the doctor; taking probiotics during and after antibiotic therapy; and discontinuing antibiotic therapy earlier than recommended by the doctor if the child feels better (Table 4). A study conducted in a group of almost 140,000 patients in Denmark to identify the socioeconomic factors that increase the risk of antibiotic use showed that parental education was the most statistically significant risk factor for unjustified antibiotic therapy (23).

It can be assumed that all of the factors presented here contribute to the more frequent use of antibiotics in children residing in rural areas and in towns with <10,000 inhabitants (*p* < 0.01). A doctor's behavior while giving medical advice can have a great impact on a patient's subsequent actions. It has been demonstrated that prescribing an antibiotic for the symptoms of respiratory tract infections significantly increases both the risk of another medical visit and the expectation of obtaining another prescription for an antibiotic (35). Previous studies indicated that patients who expected to be prescribed an antibiotic during a medical consultation for an URTI were three times more likely to receive it, even if there were no indications for such therapy. The expectations and pressure related to the hope of using an antibiotic are among the most important predictors of the type of therapy used (36, 37). Research in the United Kingdom showed that there was an increased risk of inappropriate antibiotic prescription if the attending physician was older than 40 years, whereas residents who specialized in family medicine, internal medicine, and pediatrics, and who worked in primary practice, especially those supervised by specialists and attending small patient populations, were more wary of prescribing inappropriate antibiotics (38). Our study did not consider the age of the physician or the number of patients in the population of the primary care facility. A US study in a group of 2,005 physicians showed that doctors prescribing more antibiotics are likely to be specialists other than pediatricians and further from medical school training (39). This underlines the role of education in this field not only for parents but also for the doctors themselves. We also demonstrated that the location of the practice had no significant impact on the rates of antibiotic therapy.

Data from the Ministry of Health and the Supreme Medical Chamber have shown that the average age of doctors in Poland is relatively high, with the majority of those in practice being 55–65 years (40). Our study suggested that their level of knowledge was an important factor influencing the decisions made by GPs to use antibiotics more often in rural children.

Overuse of antibiotic therapy is an important clinical problem, which increases the rates of microbial resistance (41, 42). New guidelines and studies continue to be developed to standardize the symptomatic and causal treatment of respiratory tract infections (6, 12–15, 43, 44). Data on the use of a delayed antibiotic prescribing strategy for respiratory tract infections have shown that it significantly reduces the frequency of antibiotic use, with no negative effects in terms of an increased number of complications being associated with this treatment approach (45). This type of management has already become a standard in the treatment of acute otitis media, with guidelines developed for patients both under and over 2 years of age, taking into account the relevant risk factors (1). Understanding the risk factors that significantly increase the probability of using antibiotics, including social and economic factors, and improving the knowledge held by parents of children with URTIs about antibiotic therapy, can effectively reduce the risk of unnecessary treatment and undesirable symptoms, thereby reducing the number of antibiotic-resistant strains. For this reason, it is crucial to provide continuous training for primary care physicians and education for both parents and patients.

## Conclusions

This study showed that selected environmental factors and the associated parental behaviors influenced primary care physicians' decisions to prescribe antibiotics to children with URTIs. The level of parental knowledge about the principles of antibiotic therapy was a significant factor contributing to primary care physicians' decisions about whether to use antibiotics in children with URTIs. There is a need for ongoing training of primary care physicians and education of parents/guardians.

## Data Availability Statement

The raw data supporting the conclusions of this article will be made available by the authors, without undue reservation.

## Ethics Statement

Ethical review and approval was not required for the study on human participants in accordance with the local legislation and institutional requirements. Written informed consent to participate in this study was provided by the participants' legal guardian/next of kin.

## Author Contributions

ŁD, AK, AM, and JP-P: study design. AK, DK, and JP-P: data collection. ŁD, MD, and JP-P: data analysis and interpretation. KŚ, ŁD, AK, MD, AM, MP-P, PW, DK, and JP-P: manuscript preparation and critical revision. All authors contributed to the article and approved the submitted version.

## Conflict of Interest

The authors declare that the research was conducted in the absence of any commercial or financial relationships that could be construed as a potential conflict of interest.

## Publisher's Note

All claims expressed in this article are solely those of the authors and do not necessarily represent those of their affiliated organizations, or those of the publisher, the editors and the reviewers. Any product that may be evaluated in this article, or claim that may be made by its manufacturer, is not guaranteed or endorsed by the publisher.
